# Fictional narrative as a variational Bayesian method for estimating social dispositions in large groups^☆^

**DOI:** 10.1016/j.jmp.2019.102279

**Published:** 2019-12

**Authors:** James Carney, Cole Robertson, Tamás Dávid-Barrett

**Affiliations:** a Brunel University London, Gaskell Building G29, Kingston Lane, Uxbridge UB8 3PH, UK; b Center for Language Studies, Radboud University, Netherlands; c Department of Experimental Psychology, University of Oxford, Woodstock Rd, Oxford OX2 6GG, UK; d Universidad del Desarrollo, Facultad de Gobierno, CICS, Av. Plaza 680, Santiago de Chile, 7610658 Chile; e Trinity College, University of Oxford, OX1 3BH, Oxford, UK; f Population Research Institute, Väestöliitto, Kalevankatu 16, Helsinki 00101, Finland

## Abstract

Modelling intentions in large groups is cognitively costly. Not alone must first order beliefs be tracked (’what does A think about X?’), but also beliefs about beliefs (’what does A think about B’s belief concerning X?’). Thus linear increases in group size impose non-linear increases in cognitive processing resources. At the same time, however, large groups offer coordination advantages relative to smaller groups due to specialisation and increased productive capacity. How might these competing demands be reconciled? We propose that fictional narrative can be understood as a cultural tool for dealing with large groups. Specifically, we argue that prototypical action roles that are removed from real-world interactions function as interpretive priors in a form of variational Bayesian inference, such that they allow estimations can be made of unknown social motives. We offer support for this claim in two ways. Firstly, by evaluating the existing literature on narrative cognition and showing where it anticipates a variational model; and secondly, by simulation, where we show that an agent-based model naturally converges on a set of social categories that resemble narrative across a wide range of starting points.

## 1 Introduction: the problem of large groups

Primates in general, and humans in particular, owe much of their evolutionary success to the coordinating function of complex social structures. Though many mammal species aggregate in large collectives, primates are distinctive in congregating in groups that evince multi-level hierarchies and long-term social bonds (Sutcliffe, Dunbar, & Wang, 2016). And amongst primates, humans display a particular propensity for long-term social organisation, with one of the most salient outcomes of this being trans-generational social forms like institutions, cities and nations that group together unrelated individuals in relationships of mutual advantage (Currie et al., 2016, Dávid-Barrett, 2019, Dávid-Barrett and Dunbar, 2017). Indeed, otherwise puzzling phenomena like religion and ritual have sometimes been explained as suites of adaptations that are preserved in virtue of their ability to foster large-scale, non-kin based cooperation (Cauvin, 1999, Dávid-Barrett and Carney, 2015, Norenzayan, 2013, Sosis, 2004, Sosis and Bressler, 2003, Whitehouse, 2000).

**Fig. 1:**
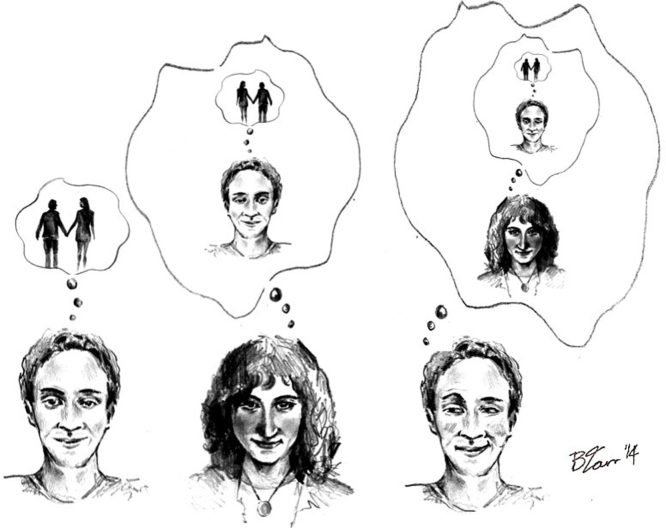
Recursive mentalising in social cognition.

**Fig. 2:**
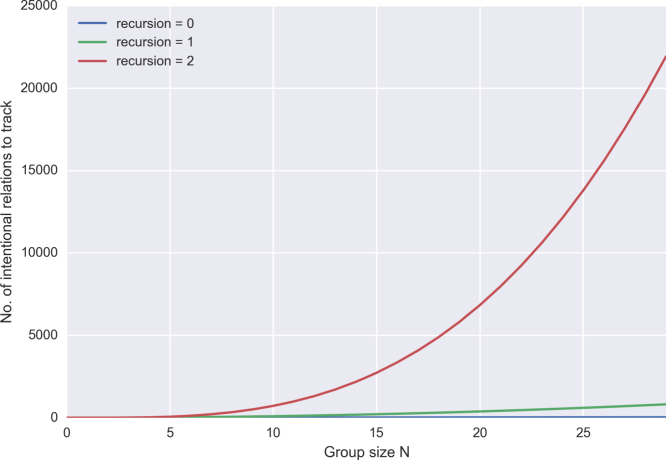
Combinatorial increase in number of recursive intentional states as group size grows.

However, for all that increased social cooperation brings collective benefits, it also imposes costs. Semi-structured groups that allow individuals to shift between roles require constant monitoring to ensure that cooperation partners are not reneging on agreements—a scenario that does not occur when cooperation roles are biologically fixed (as in eusocial insects) or exist only in trivial forms like physical co-presence (as with species living in unstructured social aggregations). Moreover, this monitoring does not centre exclusively on ego: it is also crucial that third-party relations are registered (‘What does Alice think about Bob?’), as well as recursively embedded beliefs about beliefs (‘What do I think that Alice believes concerning what Bob thinks about her attitude to him?’) (Fig. 1). As recursion of this type grows on the order of $m^{n}$ where *m* is the group size and *n* is the number of recursions, the number of intentional relations that need to be monitored will scale non-linearly with group size (Fig. 2). What emerges from this is that large groups cannot be modelled without quickly running into cognitive, mnemonic and time constraints (Dávid-Barrett and Dunbar, 2013, Dunbar et al., 2014). Indeed, the social brain hypothesis argues that the human neocortex evolved specifically to deal with this problem—and even at that, the claim is that humans have difficulty dealing with groups of more than ∼150 members (Dunbar, 1992, Dunbar and Shultz, 2007, Hill and Dunbar, 2003, Humphrey, 1976).

 In this paper, we formally model how one cultural tool – narrative – succeeds in making the cognitive and mnemonic demands of navigating complex social networks manageable. This model will build on a long-standing tradition of qualitative scholarship that interprets narrative as a cultural tool for thinking about social situations (Bruin and Haan, 2012, Gallagher, 2003, Hutto, 2008, Hutto, 2009, Mar, 2011). Where it innovates will be in giving a mathematical demonstration of how something like narrative emerges naturally when elementary coordination strategies are practised in the context of increasing group size. Our first goal in doing this will be to provide an important resource for thinking about the origin of narrative. While idealised models of cultural phenomena must always be less rich than their target object, they are nevertheless able to offer useful starting points for adjudicating between conflicting models and offer prompts for further thinking—and we hope to offer just such a starting point. Our second goal will be to elucidate why narrative has the features that it does. Narrative intrigues because it has both a cross-cultural distribution and readily identifiable features (Brown, 1991, Herman, 2002, Sugiyama, 2001); as such, it invites explanation as to why those features and not others should be visible across cultures. We shall offer this by showing how processes of group coordination converge on a set of cognitive and cultural categories that become valuable when they are not tied to real-world situations. In other words, our model will account for the tendency of narrative to lodge highly prototypical action roles in fictional worlds. Doing this, we submit, will yield important insights into the role played by fictional representations in human cognition.

We shall proceed with this agenda in three stages. Firstly, we shall give a short review of what is typically understood by narrative. This will have the orienting function of guiding the analysis with a reasonable appreciation of what narrative actually is, and support our extraction of three features in particular – prototypicality, fictionality, and temporality – as crucial to the understanding of narrative. With a view to grounding the discussion, we shall also offer a short exposition on Greimas’s (Greimas, 1983, Greimas, 1987) actantial theory of narrative, which provides a particularly instructive example of how narrative can be theorised in terms of prototypical action roles.

This second step will come with the mathematical analysis, which will blend a Bayesian variational framework with an agent-based synchrony model. The variational framework will show that the action roles typically encountered in a narrative comprise a probability distribution that approximates another, much more complicated, probability distribution: that defined over the entire set of motivations potentially animating an agent in social life. This process, we argue, reduces the dimensionality of social relations by creating a set of expectations that guide inferential processes in specific ways. However, while this certainly provides a useful framework for thinking about the function of narrative, it still falls short of explaining how narrative has the precise features that it does. To account for this, we supplement the variational Bayesian approach with an agent-based synchrony model that shows, under realistic assumptions about agent behaviour, how the action roles of narrative emerge as a stable cultural pattern that is robust against different initial conditions. In other words, the synchrony model identifies how narrative structure emerges naturally from the fundamental transactions of human sociality. Taken together, these approaches unite in theorising narrative as a cultural tool, shaped over generations of use, that supplies a process of Bayesian estimation with a set of priors to reason with. Allowing that this estimation is optimised in cognitively realistic ways, we propose that repeated encounters with items of socially relevant evidence in a given domain of action will allow for the construction of multiple narrative models that are each tailored to fit different forms of social life.

We shall pursue the final step in our approach by using our account of narrative to solve a long-standing problem in the philosophy of culture. Specifically, while counterfactual thinking has obvious benefits when it comes to thinking about the motives of other agents, it is less clear why we should be engaged by social scenarios that are explicitly announced to be false and with agents and worlds that could never come into existence. As one philosopher of narrative notes, ‘while virtually everyone consumes fictional narratives, no one … has an interest in fictional theories. There are science fiction novels, but there is no fictional science’ (Currie, 2010 p. viii). As we shall show, treating narrative as a form a variational reasoning allows for this problem to be resolved in a way that balances the costs and the advantages of entertaining fictional models of social relations.

We are aware that our stated goals are ambitious. Delivering a solution to the problem of recursive relations in social thinking while also tackling the paradox of fiction is a large burden of expectation to place on a short paper. However, our methods show that the two problems are inverse sides to the same issue, to the extent that fictionality is what allows narrative to approximate complicated social relations without needing to be made consistent with any real-world history. That is, fiction represents the decoupling of a contingent probability distribution from its antecedent conditions. To say more than this now would be to anticipate the discussion; the point is merely to indicate that our paper’s aims, though they may seem heterogeneous, are united by a deeper logic.

Finally, it is worth saying before we begin that we see our model as contributing to the wider literature on cognition and social categorisation. Though narrative is more than just a classificatory schema, it is nevertheless the case that it centres on highly prototypical roles and functions. In this, it is of a piece with other classificatory systems that make large groups easier to process by mapping them into simplifying schemas. Examples include tag-based coordination systems, where perceptual signals like accent, dress, and exacting religious observances act as markers that allow one to treat multiple cooperation partners in the same way (Cohen, 2012, Smaldino, 2017, Weber, 2009); kinship terminologies, which are proposed to reduce cognitive load by mapping multiple individuals into pre-defined categories that have a fixed set of behavioural expectations (Brashears, 2013, Chatters et al., 1994, Lakoff, 2002, Machin and Dunbar, 2016); and layer-based clustering of the ego-centred social network, such that individuals are sorted into close, intermediate, and distant contacts (Mac Carron et al., 2016, Zhou et al., 2005). By aligning narrative with these phenomena, we hope to show that, far from being *sui generis*, it forms part of a wider suite of cultural tools that help negotiate the social world—and can be best understood by viewing it in conjunction with them.

## 2 The nature of narrative

Though there is widespread agreement that narrative has a cross-cultural presence in human societies, there is less consensus on what narrative actually is (Ryan, 2007). Sociological theories of narrative see it as a highly general term that can be used to label any discourse that represents an agent or agent-like process (Geertz, 1973, Lyotard, 1984, Somers and Gibson, 1994). Linguistic theories argue for well-defined discursive or semiotic features that mark narrative or genres of narrative as distinctive forms of communication (Barthes, 1977, Lévi-Strauss, 1955, Propp, 1968, Todorov, 1971). And cognitive theories see narrative less as a concatenation of signs than a pragmatic stance or a mode of cognition or that is oriented towards the logic of motives over the logic of propositions (Bruner, 1991, Fludernik, 2004, Herman, 2013, McAdams and McLean, 2013).

In the face of such a diversity of opinion, it is tempting to regard the concept as incoherent and dispense with it as a worthwhile category. However, recent work on the concept of ‘narrativity’ offers a useful way to frame this disagreement. This maintains that narrative is a matter of degree as much as kind, so it is less useful to talk about *whether* or not an item is a narrative than the *extent* to which it is a narrative (Fludernik, 2004, Herman, 2002, Prince, 2004). By this view, a rigorous definition of narrative matters less than an identification of the kinds of features that identify a given representation or mode of cognition as being narrative-like. These features would need to be specific enough to distinguish narrative from other forms of discourse, yet of sufficient generality to allow a broad range of variability and grey areas. In this regard, we propose the three traits of *temporality*, *prototypicality* and *fictionality* as useful benchmarks for what a model of narrative needs to take account of.

That narratives are temporal is perhaps their most salient feature. Indeed, some of the most important works of narratological scholarship have taken up the relation between narrative and time (Ricoeur, 1985). Here, the idea is usually that narratives, as the representation of agent-like entities acting in time, are expressive of very fundamental intuitions about how action is shaped by temporal considerations. We do not disagree with this view, but nuance it by placing an emphasis on predictability. For us, the projection of categories into the hypothetical or actual future is essentially a predictive act rather than merely a classificatory one. In this way, narratives do not correspond to descriptions of states of affairs; they are instead probabilistic predictions of what future states of affairs might be like, given past and present experience and knowledge. An early model of this projection of the past into the future is provided by Heidegger (2008). More recently, a rich literature has emerged that looks at how narrative functions as an autobiographically focused tool for consolidating memory and negotiating trauma (Fivush et al., 2011, Sales et al., 2013); at a cultural level, postcolonial, gender, and critical race theories of narrative discuss how narratives can function to express subaltern and marginalised identities as ongoing projects that link historical experiences to future expectations (Bhabha, 1990, hooks, 1991, Sommer, 2007, Young, 2018). Though few of these perspectives are explicitly cognitive in orientation, the cultural formations they explore evince the different ways in which thinking about the future can mesh with socio-cultural realities. Necessarily, this shows up narrative as powerful tool for actual or aspirational prediction, and the deep relation between prediction and narrative temporality is thereby disclosed.

These thoughts on temporality lead naturally into the topic of prototypicality. Specifically, there is an intuitive sense that a narrative should be ‘about’ something beyond itself. Though most narratives involve the evocation of particulars, ‘the particulars of narrative are tokens of broader types’ (Bruner, 1991 p. 6). At the most prosaic level, this is visible in the two-way interaction between narratives and the cognitive frames, scenes, and scripts that structure thinking and action in the real world (Fludernik, 2004, Herman, 2003). Two-way, because while narratives are often explicitly designed to communicate these structures when directed at children, they usually assume knowledge of them when directed at adults (Herman, 1997, Hutto, 2008). In this sense, the action sequences embedded and evoked in narrative have a character of typicality that problematises their location in any specific time and place. However, it should also be recognised that the typicality of narrative discourses is not the same as saying that narratives merely articulate a set of categories. Central to the experience of narrative is the expectation of a breach with established norms, which initiates the temporal movement of the narrative (Bruner, 1991, Propp, 1968). Being expected, this breach is typical, but its presence is enough to distinguish narrative from a mere taxonomy and make it adequate to the normative considerations that enter into human actions.

At a more abstract level again, similar considerations enter into the role of symbolism on narrative. Here, the emphasis falls on narrative tokens as vehicles for culturally sanctioned schemes of interpretation by way of devices like allegory, fable, and participation in wider hermeneutic traditions (Barthes, 2002, Gadamer, 2004, Ricœur, 1995). Though the extraction of these latent meanings often requires considerable interpretive work, it presupposes the idea that narratives are saturated with more meaning than their denotative content indicates.

However, it is principally with respect to the description of agent action and psychological states that the notion of prototypicality takes on its most important meaning. Characters in narrative are always, to some degree, typical: in the simplest of cases, this can correspond to the allegorical equating of specific traits with specific characters in a one-one correspondence; more common representations show characters moving between different mental states in processes of transformation—often as a result of the processes of breach and violation that are concomitant with narrative. What is visible in every case is the structured nature of the representation. Whether the structure resides in the character-value identity or the types of transformations that characters undergo, it has predictable forms that recur across narratives. Unsurprisingly, narrative scholarship has expended considerable effort in cataloguing these structures. Starting with Vladimir Propp’s *Morphology of the Folktale* (1968), there is a sustained literature that seeks to identify the roles that recur across narratives, the action schemas these roles are embedded in, and the cognitive operations that underwrite both roles and actions (Barthes, 1977, Fludernik, 2004, Gerrig and Allbritton, 1990, Propp, 1968, Todorov, 1971).

As the derivation of the variational model of narrative in the next section will be much clearer with a concrete model of narrative prototypicality in mind, we shall now offer a short exposition on one of these models—namely, the actantial model of Greimas, 1983, Greimas, 1987. It should be noted that we do not do so out of any conviction that Greimas is right where others are wrong, but because it provides a succinct illustration of narrative prototypicality with respect to actional and psychological dispositions that is particularly convenient for the purposes of our exposition. Other models would do just as well; no normative judgment is implied by our not considering them—our aim is only to illustrate how a typical narrative model succeeds in reducing social complexity to a small number of roles and prototypical situations.

In terms of detail, the vehicle of prototypicality in Greimas’s model is the concept of an *actant*. Though the term derives from linguistics (Tesnière, 2015), it has taken on its primary meaning as a designator for generic action roles that can exist in the absence of a supporting subjectivity, much as grammatical categories can depict actions while evoking the psychological motivations that enter into them (Latour, 2004). In this regard, Greimas proposes that narratives consist of six actants divided into three pairs: *subject* and *object, sender* and *receiver*, and *helper* and *opponent*. The core relation amongst these roles is argued to be the relationship of desire, want or need between subject and object, where the subject desires some person, thing or state of affairs – the object – that it does not have direct access to. This desiring relation between subject and object is in turn mediated by the relation of *alliance* (or hostility) between the subject, helper and the opponent. Finally, these *practical* relations of desire and alliance are then both overarched by the *normative* relation of legitimation between the sender and receiver, insofar as the sender authorises the subject in its actions, the good of which accrue to the receiver. (In all cases, it should be noted that a character’s actions can be informed by several actants, just as a single actant can inform the role of several characters.)

An example will make these ideas clear.In Arthur Conan Doyle’s Sherlock Holmes narratives, Holmes is clearly the subject, with the object being simultaneously to solve a crime and relieve himself of boredom (Holmes uses cocaine to distract himself when there are no crimes for him to solve). The helper corresponds to Watson, as well as Holmes’s powers of reasoning and knowledge of forensic arcana; the opponent is criminal ingenuity. The sender is the petitioner who comes to the detective and authorises him to act in the name of justice; the criminal justice system that punishes the criminal for the benefit of the general public, embodied through representatives like Lestrade, is the receiver. Moreover, according to Greimas, these roles are not just visible in fictional narratives, but also in narratively mediated ideologies and value systems in areas like politics and religion (Table 1).

These considerations lead naturally to the third trait of narrative we are concerned with—fictionality. By exploiting roles that are abstract enough to encompass the nature of action in general while being concrete enough to attract psychological ascriptions, narratives clearly evince prototypicality. However, while prototypicality is not the same as fictionality, there is a sense in which both move away from the locating of narrative events and items in the actual world. In the case of prototypicality, this comes by way of representations that are consistent with several possible worlds; in the case of fiction, the emphasis falls on representations that are consistent with a different possible world. And, like prototypicality, fictionality is visible across the repertoire of narrative production. This much is already visible in the counterfactual orientation of the mythological, folkloric, and literary record. However, there is also a sense in which even narratives about real-world events are quasi-fictions. On the one hand, there are no linguistic or discursive features attaching to factual narrative that systematically distinguish it from fictional narrative (Genette, 1990); on the other, the inexhaustible character of the actual world means that any successful form of narrative representation must necessarily involve condensations, elisions, and stylisations that approximate towards fiction. Indeed, this latter tendency is particularly visible in narratives about historical figures that are frequently re-told, where fictional motifs and flourishes often come to displace the factual content (Powell, 2010). If so, fictionality is woven through narrative, and its presence is matter of degree rather than kind.

**Table 1:** Distribution of actantial roles across several narratives and narrative ideologies.

Actant	James Bond	Lord of the Rings	Christianity	Marxism
Subject	Bond	Frodo, hobbits	Mankind	Proletariat
Object	Protection of democracy	Destruction of the Ring	Salvation	Dictatorship of the proletariat
Helper	Q, Bond girl, Felix Leiter	Fellowship of the Ring	Christ	Communist movement
Opponent	USSR, SMERSH, terrorism	Sauron, Saruman, despair	Satan	Capitalism, bourgeois morality
Sender	M, Crown	Council of Elrond	Eve, original sin	Material dialectic
Receiver	British public, western world	Mortal civilisation, Middle Earth	The saved elect	The future

Where fictionality poses a problem that prototypicality does not, however, is in the fact that it commits consumers of narrative to counterfactual worlds. While there is an obvious value in being able to reason counterfactually about real-world social situations (‘If *X* were the case, how would *A* react?’), this is not the case the vast majority of narrative fictions. Here, counterfactual reasoning is invited about persons that do not exist in scenarios that could never come about (Currie, 1990, Currie, 2010, Radford and Weston, 1975). And yet, fictional narratives are a cross-cultural universal (Brown, 1991, Sugiyama, 2001). Though this problem is not typically framed in evolutionary terms, this is where the crux of the issue lies. Specifically, memory, attention and comprehension all degrade rapidly in the face of competing stimuli (Borst et al., 2010, Sigman and Dehaene, 2005), so the cultivation of impossible counterfactual representations impinges on the processing of other more useful and ecologically relevant information. Thus, fictions are not just redundant; they seem to be positively disadvantageous when it comes to negotiating the real world—a reality that would have been even more pressing in the time- and energy-scarce Palaeolithic, when it is likely that cultural technologies such as narrative first emerged (Pinker, 2007).

The general point to emerge from these considerations is that any viable theory of narrative fiction should engage with at least the issues of temporality, prototypicality and fictionality, and ideally offer some insight into the problem of fictionality. No doubt, a case can be made for it to be able to do more than this; nevertheless, we argue that they provide a plausible floor for the explanatory power of a theory of narrative. In the next section, we shall develop such a theory, where both prototypicality and fictionality are explained as the outcome of an agent-based synchrony process lodged in a predictive Bayesian variational model that enacts a form of temporality.

## 3 Formally modelling narrative

Our core claim, following others, is that narrative is a cultural prosthesis that aids in the processing of large group sociality. Necessarily, qualitative appreciations of narrative can go some distance towards showing how this might occur. However, such appreciations must remain speculative unless they can be integrated with wider frameworks of human cognition and behaviour. In this section, we shall show that two such frameworks – probabilistic reasoning and behavioural synchrony – can be used to derive a formal model of narrative. Consistent with our wider aims, this model should show how narrative reduces the cognitive load associated with modelling social relations by creating a smaller set of action categories that can be used to engage with actual and potential social encounters. In this way, narrative fiction will be shown to be a crucial tool for navigating the social world; moreover, as we shall see, our resulting model is consistent with the qualitative considerations explored in the preceding section.

To begin, it is worth putting a clear framework on the problem. Suppose that an individual exists in large group with *m* members, such that she does not have information about the majority of group members but is nevertheless obliged to engage in repeated social interactions with them. In such a scenario, the problem comes with inferring the likely motivations of other group members on the basis of whatever evidence, *X*, that may be available in a given encounter. (Motivations, in this context, refer to situational reasons for action that can change from one scenario to the next, rather than stable personality traits. As such, they anticipate actantial roles.) That is, for a probability distribution of possible dispositions $Z=Z_{1},\ldots ,Z_{n}$, she needs to establish the conditional probabilities $P\left(Z_{i}|X\right)$—in other words, the likelihood of motivation $Z_{i}$ given the presence of evidence *X*. This means establishing the posterior probability as defined by Bayes’ formula:

(1) $$P\left(Z|X\right)=\frac{P\left(X|Z\right)P\left(Z\right)}{P\left(X\right)}$$

As the denominator probability *P*(*X*) is calculated in relation to both the marginal probability of *X* given each $Z\in Z_{1},\ldots ,Z_{n}$ and the probability $P\left(Z_{i}\right)$ for 1 ≤*i* ≤ *n*, the total distribution is defined by Eq. (2), so long as the motivations are assumed to be mutually exclusive. This is likely to be untrue in practice: human agents are almost always actuated by several motives, and much of social cognition involves balancing one against another (‘I need to get what I want consistent with remaining polite’.) However, allowing for this will increase the complexity of the formalism without clarifying the principles involved; more importantly, it may well be that the efficiency in assigning an agent just one motive outweighs the loss of accuracy in assigning several motives.

(2) $$\overset{n}{\underset{i=1}{\sum }}\frac{P\left(X|Z_{i}\right)P\left(Z_{i}\right)}{P\left(X\right)}=1$$

**Fig. 3:**
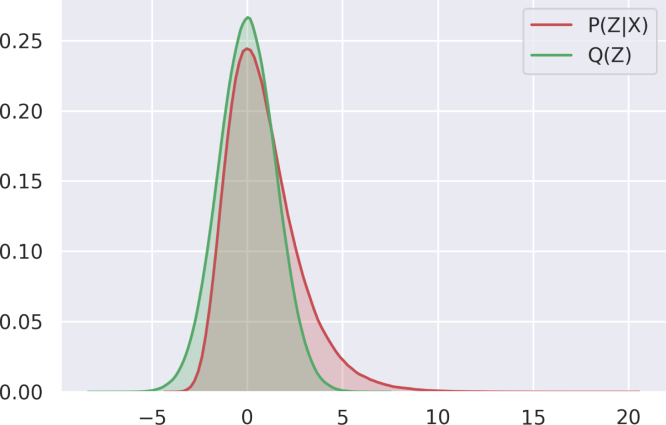
Estimation of complicated distribution P(Z|X) by simpler distribution Q(Z).

But here a further difficulty arises. When motivations are restricted to first order mental states, *m* is equivalent to group size and it may be already potentially infeasible to calculate the posterior probability. However, it becomes entirely intractable with the addition of recursive mental states, due to the combinatorial explosion in the number of motivations (and motivations that respond to other motivations) that need to be tracked. As this problem cannot easily be solved by direct calculation, the best solution is to approximate the posterior probability. One method for doing this – variational Bayesian estimation – has emerged in recent scholarship as an important model for how organic and cognitive processes estimate environments that cannot be exhaustively sampled (Devaine et al., 2014a, Devaine et al., 2014b, Friston, 2010, Sanborn, 2017). In practice, these methods involve approximating the intractable distribution P(Z|X) with another distribution *Q*(*Z*) (Fig. 3), such that

(3) $$P\left(Z|X\right)\approx Q\left(Z\right)$$

The idea is that, so long as the estimating distribution *Q*(*Z*) is simpler than P(Z|X), it becomes possible to approximate the more complicated distribution by defining a divergence function, which measures the difference between the two distributions. Naturally, finding a *Q*(*Z*) that minimises this divergence will result in a more accurate approximation to P(Z|X). Typically, this is calculated using the Kullback–Leibler divergence ($D_{KL}$, also known as relative entropy), which outputs a value of zero when the distributions are identical:

(4) $$D_{KL}\left(Q∥P\right)=-\underset{Z}{\sum }Q\left(Z\right)\log\frac{Q\left(Z\right)}{P\left(Z|X\right)}$$

However, as the quantity P(Z|X) is precisely what we are trying to estimate, the divergence is algebraically rearranged to an expression of the form:

(5) $$\log P\left(X\right)=D_{KL}\left(Q∥P\right)-\underset{Z}{\sum }Q\left(Z\right)\log\frac{Q\left(Z\right)}{P\left(Z,X\right)}$$

As the quantity *log P*(*X*) is fixed, it follows that increasing the value of the summation on the right-hand side – technically, the variational free energy – will force $D_{KL}$ towards zero (as a measure of divergence, it cannot be less than zero). Thus, the task of minimising the difference between P(Z|X) and its approximation *Q*(*Z*) becomes finding the distribution *Q*(*Z*) that does this most effectively.

**Fig. 4:**
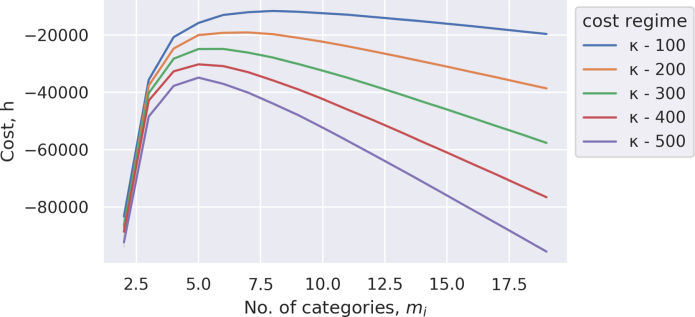
Payoff varies by category number and cost of having higher number of categories.

It is at this point that the synchrony model enters the picture. Unlike many instances of variational Bayes, where very little is known about the latent variables, our knowledge of human cognition means that are established external constraints that will prevent *Q*(*Z*) from ever truly approximating P(Z|X); these, naturally, will affect the values $D_{KL}$ can take, and thus determine the point at which the optimisation procedure stops. To see how this occurs, let us return to the motivating scenario.

From an evolutionary perspective, our agent’s problems are motivated by the need to optimise two countervailing quantities. On the one hand, she needs to minimise the cognitive load associated with processing resources and memory costs in assessing motivation relative to social evidence; on the other, she is required to maximise the payoff that comes from engaging with other agents (with payoff including the resources saved by avoiding hostile agents). Thus, her selection of an approximating distribution *Q*(*Z*) is not solely determined by a low value for $D_{KL}$; she needs to select the lowest value of $D_{KL}$ consistent with the highest payoff. The first way in which this can be done is with respect to the number of items in $\left\{Z_{1},\ldots ,Z_{n}\right\}$. A small number of items will impose a lower cognitive load but increase the possibility of precision errors in matching agents to dispositions; a large number will increase precision but impose a higher cognitive load. How do we establish the optimum ratio of cognitive load to precision? The synchrony model allows us to establish a possible answer by way of a simulation mechanism.

Let us assume that every agent *i* has a true nature, ϕ, that is signalled by the social evidence, *X*. For the purposes of the synchrony model, the possible values of ϕ can be thought of as uniformly distributed on the unit circle between 0°and 360°. As there will be a finite number of these true natures, representing them as occurring in the unit circle makes it easier to randomly sample from them roulette-wheel style than would be the case, say, if they were represented on a line:

(6) $$\phi ∼U\left(0{}^{\circ},360{}^{\circ}\right)\forall i$$

In assessing motivations on the basis of social evidence, the key task for each individual agent becomes that of assigning a category to every other agent in an encounter. For this purpose, each agent *i* has a set of categories $Z_{i}$. Let $m_{i}$ denote the number of categories in $Z_{i}$. Formally, $Z_{i}$ is generated by selecting positions in the unit circle, such that positions correspond to types:

(7) $$Z_{i}\equiv {\left\{z_{i,s}=\alpha_{i}+s.\frac{360}{m_{i}}\right\}}_{s=1}^{m_{1}}$$

Here, α is a random starting point for generating the categories and *s* is a category counter. Thus, for $m_{i}=4$, the result is the unit circle being divided into four quadrants. Given the set $Z_{i}$, agent *i* then categorises all others in the network such that they are put into the category to which their respective types fit best:

(8) $$f_{i.j}=\underset{z_{i,s}}{\text{argmin}}{\left\{\phi_{j}-z_{i,s}\right\}}_{s=1}^{m}$$

That is, the category choice that produces the least error is the one chosen. It is at this point that questions of cost and payoff enter the picture. The payoff function, *h*, can be defined in terms of a cost parameter, κ, and the number of categories, $m_{i}$, in expression (9):

(9) $$h\left(\kappa ,m_{i}\right)=h_{\omega }-\overset{n}{\underset{j=1}{\sum }}{\left(\phi_{j}-f_{i,j}\right)}^{2}-\kappa .m_{i}$$

The assumption here is that ω will set a fixed value of *h* for each implementation of the model; the payoff will be given by this term minus the quadratic error in predicting the types of other agents, and minus the cost parameter by the number of agent categories. Assuming $h_{\omega }=0$ and κ is fixed for all agents, Fig. 4 shows the dual effect of the cost of the prediction error and the maintenance cost of large number of categories on payoff. Trivially, the increase in the number of categories decreases the prediction error. However, given that this also increases the cognitive costs, captured by $m_{i}$, the optimum number of categories needed to model all the socially relevant beliefs, *n*, is *n*-1 only if the cognitive cost, κ, is sufficiently small.

 With the payoff regime established, the question becomes what specific number of categories, $m_{i}$, delivers the best payoff relative to costs. This can be established by way of an evolutionary approach, such that the agent with the lowest payoff ‘inherits’ the number of categories of the agent with the highest payoff. Formally:

(10) $$m_{i,t+1}=\left\{\begin{array}{c} \underset{m_{j,t}}{\text{argmax}}{\left\{h\left(m_{j,t},\kappa \right)\right\}}_{j=1}^{n}\;\\ \;\;\mathrm{if}\;h\left(m_{j,t},\kappa \right)=\underset{m_{j,t}}{\min}{\left\{h\left(m_{j,t},\kappa \right)\right\}}_{j=1}^{n}\;\\ m_{i,t}\;\text{ otherwise}\; \end{array}\right)$$

**Fig. 5:**
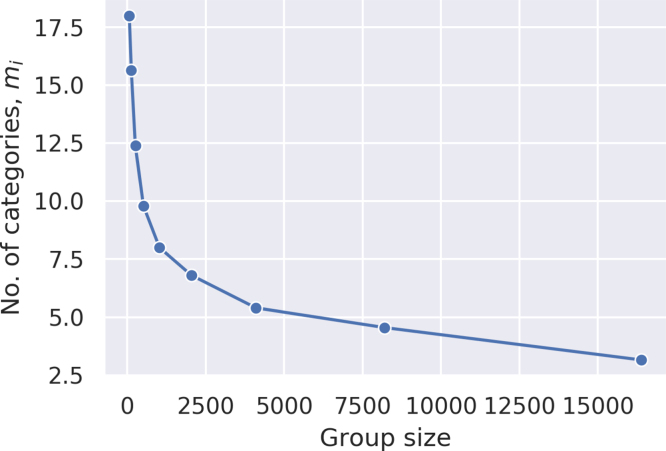
Evolved number of categories.

The algorithm defined by these constraints should converge on the specific value of $m_{i}$ that determines the optimum number of categories for all agents. Fig. 5 shows the results of doing this. As can be seen, the evolved number of categories is dependent on the cost of maintaining these categories. (This curve corresponds to the maximum points on the curves depicted in Fig. 4.)

**Fig. 6:**
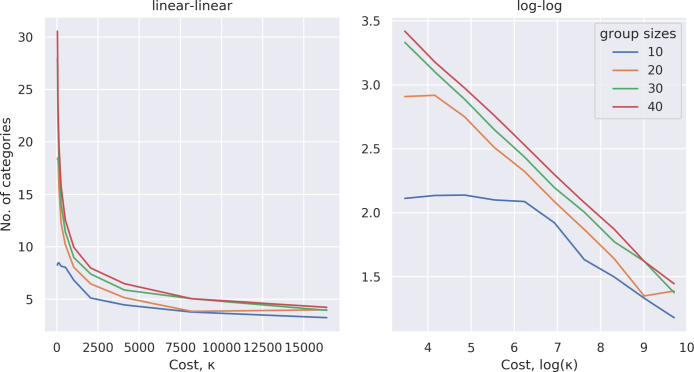
Evolved number of categories as a function of category cost and optimal number of categories. Left panel: linear–linear scale. Right panel: log–log scale.

Given the importance of group size to our motivating question, it is crucial that we ask how the relationship between the cost of the categories and their optimal number changes with group size. Fig. 6 shows the results of simulating this. Notice that the m*(κ) function is exponential, except for the upper limit of categories due to the group size. (The category number cannot exceed *n*-1.) The consequence is that when having categories is cheap, the optimal number of categories will increase rapidly with the group size. However, when the cost of having categories is high, then the number of categories ends up essentially the same independent of group size. As the number of necessary categories increases combinatorically in a linearly increasing population, even a negligible cost per category quickly becomes unsustainable. Thus, though the specific value of $m_{i}$ that emerges will depend on the exact convergence criteria, it seems that a value $m_{i}$ between 4 and 7 represents the optimal number.

**Fig. 7:**
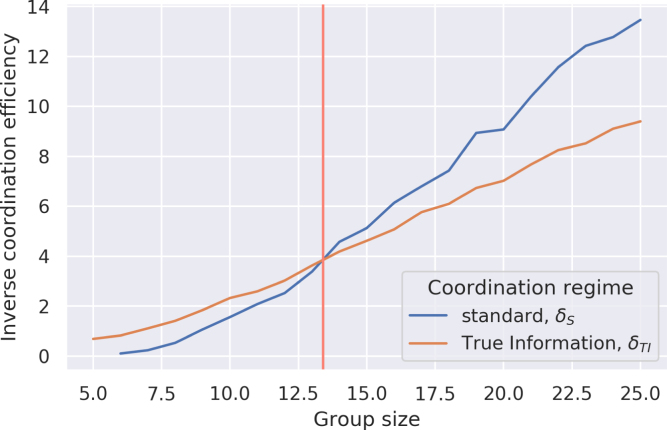
Improved group coordination with True Information as group size increases. The vertical line marks the threshold where the TI regime becomes more efficient than standard coordination.

Returning to the motivating framework, this suggests that the variational estimation distribution *Q*(*Z*) should be defined over a set $\left\{Z_{1},\ldots ,Z_{n}\right\}$, such that 4≤n≤7. Inevitably, this poses the question of what categories comprise the members of this set. In this connection, we have elsewhere used the synchrony model to show how normative standards spread across networks (Dávid-Barrett & Carney, 2015). We shall not reprise the analysis here, but it uses the same synchrony methodology to show that in networks over a certain size, collective coordination is improved by the presence of an agent who does *not* alter her position in the synchrony update process (Fig. 7). This figure – which we term the ‘True Information’ (*TI*) – essentially acts as the point around which normative standards can coalesce; thus, they accelerate collective coordination. Though our model indicates that *any* coordination standard held by the *TI* is more efficient than *no* coordination standard, it is plausible that standard selection effects would reward those standards that are best adapted to the actual social environment. If so, it should be evident how this result can be applied to categories for social cognition. Specifically, in any evolutionary competition between sets of social categories that can match up to actual social categories with greater or lesser degrees of fidelity, the adaptive value of those categories that match up well represents a type of inertia in face of coordination pressures. Necessarily, those well-adapted categories will come to play role of the *TI* in our model and are more likely to spread across the network as a normative standard.

Putting all this together, what thus emerges is a synchrony model lodged in a variational Bayesian framework that is capable of generating a prototypical action scheme that approximates existing models of narrative. In the first instance, the 4 ≤ *n* ≤ 7 number of categories generated by the synchrony model matches up with the 6 categories of the actantial model; in the second, this number emerges from a large number of different starting points, thereby explaining one element of the cross-cultural similarity of narrative structure. By this view, fictional narrative becomes a cultural expression of an underlying cognitive strategy for dealing with the cognitive costs of living in large groups. Though this may seem to reduce narrative to a second-order reflection of a more fundamental mode of cognition, it is likely that the relation also runs in reverse, such that exposure to narrative during development plays a causal role in social cognition. To this extent, our model is consistent with feedback and feed-forward models of cultural processes that see evolutionary, developmental, and cultural processes as reciprocally interacting (Friston and Frith, 2015, Henrich and Henrich, 2007, Laland et al., 2000).

## 4 Conclusion: The variational model and the paradox of fiction

By way of conclusion, we propose to return to the paradox of fiction. This, it will be remembered, centres on the problem of why we should invest cognitive effort in processing scenarios that we explicitly know to be untrue. This is distinct from counterfactual reasoning, where the emphasis falls on taking existing states of affairs and exploring how they may eventuate when conditioned on *likely* or possible events. While this may seem to allow for fiction as a vehicle for counterfactual thinking, the ease with which fiction depicts implausible or impossible scenarios makes this unlikely. So, the question remains: How do we account for the cognitive attraction of fiction?

We propose here that the variational model of narrative provides a solution to this problem. The thrust of our argument has been that narrative represents a probability distribution that approximates a more complicated distribution, with the latter distribution being conditioned on social evidence. The value of the simplified narrative distribution is that it makes predictive reasoning about motivation in social groups more efficient (if less accurate). However, the means by which it does this – converting the conditional distribution P(Z|X) into the independent distribution *Q*(*Z* ) – removes the evidential constraint on application of the estimated model.

It is here that the relevance of these considerations for the paradox of fiction becomes visible. If we are correct, the value of fictional narrative lies precisely in the fact that it is not tied to a specific item of evidence in actual experience: its cognitive advantage derives from its defining a free-standing estimation of social dispositions. Were it to be conditioned on here-and-now social situations, it loses the analytical traction it provides on any other form of evidence and cannot be re-used as a social estimator across different situations. In fact, there is an identifiable tendency across different forms of narrative fabulation to explicitly remove narrative models from even the possibility of an encounter with actual evidence. The overwhelming majority of narratives, for instance, are located at extremes of spatial or temporal distance, and the frequent inclusion of unlikely or impossible events ensures that no confusion with actual social realities is possible (Carney, 2017). In those cases where there *is* an attempt at verisimilar evocation of social reality, it is usually partnered with stylistic defamiliarization or robust cultural conventions determining what should be counted as fiction (Davis and Thomas, 2016, Randall, 2010, Watt, 2001). Viewed through this lens, the costs of entertaining fiction become explicable as a means of gaining access to versatile social models that are not locked into historically specific situations. In this, we see a parallel with cosmological and religious systems of explanation, which similarly seek to quarantine estimated explanatory variables from all but the most tenuous connections with reality (Atran, 2002, Boyer, 2001, Carney and Dávid-Barrett, 2017, Dávid-Barrett and Carney, 2015). Equally, this view argues that the blurring between fictional and factual narratives alluded to earlier corresponds to the degree to which a narrative admits falsification. Genres like the autobiography readily admit falsification and thus have a lower cognitive value for modelling prototypical social exchanges; genres like fantasy or mythology cannot be falsified at all, and thus have a higher thematic value.

If this is so, what emerges at the most general level is that the variational model accounts for the prototypicality of narrative, the association with temporality, and narrative’s persistent association with fictionality. Each feature emerges as a device that simplifies processing future social relations by extracting generic features of action and isolating them, to greater or lesser degrees, from falsification. Necessarily, this view does not do sufficient justice to the empirical richness of narrative, with the result that it is probably less useful to think of there being one universal type of narrative than a patchwork of local variations on a single framework. Viewed through this lens, the variational process achieves its results by optimising over classes of evidence and constructing domain-specific narrative models. This would generate a suite of narrative models that are based in different estimating distributions, *Q*(*Z*), for different forms of social life, such that these models define ‘the narrative practices that characterise most of our everyday encounters with others’ (Gallagher, 2011 p. 25). The most obvious way that this can be seen to occur is with respect to narrative genres, where different types of actions attract different distributions of probabilities across actants (Greimas, 1983). However, there is also a substantial literature on narrative and identity that equates narrative with forms of life that, in different ways, reflect and shape the traditions, events, and traumas that make up our social and cultural transactions (Carney, 2012, Fanon, 1963, Kleinman, 1988, Kreizer, 2004, MacIntyre, 1984, McAdams, 2006, Ricoeur, 1991). By this view, the proliferation of narrative forms becomes explicable as different sets of priors that help account for different way of being in the world—in other words, as ‘multiple generative models that can be deployed depending on the situation in which we find ourselves, or the person that we are communicating with’ (Friston & Frith, 2015 p. 140).

Our final thought is to volunteer variational Bayesian methods as a framework for the analysis of cultural categorisation more generally. Here, we have restricted ourselves to narrative, with a small allusion to counterfactual thinking. It should be evident, however, that any cultural exercise that has categorisation at its core can also be understood as an estimation of hidden variables. Much has been written on this topic already in cognitive science (Boyer and Ramble, 2001, Sperber, 1996, Sperber, 2006), but such expositions would also benefit from being integrated with a more general understanding of how human culture, no less than cognition, uses inferential strategies to make sense of the world. For this, a variational Bayesian framework offers the clear advantage of a mathematically rigorous model that identifies cultural categories as rational responses to the inherent uncertainty of social and natural life.
